# A systematic review of the impact of COVID-19 on the game addiction of children and adolescents

**DOI:** 10.3389/fpsyt.2022.976601

**Published:** 2022-08-18

**Authors:** Tae sun Han, Heejun Cho, Dajung Sung, Min-Hyeon Park

**Affiliations:** ^1^Department of Psychiatry, Wonju College of Medicine, Yonsei University, Wonju, South Korea; ^2^Department of Psychiatry, Eunpyeong St. Mary's Hospital, Catholic University of Korea, Seoul, South Korea

**Keywords:** children, adolescent, game addiction, internet gaming disorder, COVID-19

## Abstract

**Background:**

During the COVID-19 pandemic, it is reported that children and adolescents who are socially isolated experience high levels of stress and various mental health problems. At present, little research has been done to collect previous studies that focused on game addiction in children and adolescents during the COVID-19 pandemic. In this research, we aimed to investigate the prevalence of gaming disorder during COVID-19 in children and adolescents and the various factors experienced by children and adolescents that affected gaming disorder.

**Methods:**

We searched PubMed, Embase, PsycINFO, and Cochrane on 5 May 2021 to identify relevant literature. We extracted the prevalence estimates of game addiction from the studies to measure the global prevalence of game addiction. Then, we found the answers to the questions raised and synthesized them into several themes.

**Results:**

We identified 2,609 articles. Among them, studies that were not related to the topic, duplicated, and that did not meet the selection criteria were excluded, and 18 studies were selected. We rated most of the studies as moderate, and a few were low, and high. A majority of studies found an increase in game usage time and game addiction score during the COVID-19 pandemic. Some children and adolescents in emotional pain play games to communicate with their peers. Regarding parenting, violent parenting and the absence of parental supervision increase levels of game addiction in children. Gaming disorder was caused by the impact of COVID-19 in a vulnerable group with predisposing factors such as depression, anxiety, and attention-deficit/hyperactivity disorder. Adolescents and males scored higher on a game addiction scale, although we could not find any quantitative correlations due to the heterogeneous scales used for gaming addiction.

**Conclusions:**

During the COVID-19 pandemic, isolated children and adolescents reported increased gaming hours as a result of coping with their psychological pain and avoiding social isolation. Their parents, who should provide proper supervision, also failed to provide appropriate support due to the stress caused by the pandemic. Mental health providers should educate children, adolescents, and their guardians on alternative ways to relieve stress and help parents effectively control their children's usage of games.

## Introduction

In 2019, an acute respiratory disease called COVID-19 was discovered in Wuhan, China, and spread rapidly across 20 countries worldwide. In January 2020, the World Health Organization (WHO) declared a public health emergency and later declared a pandemic in March 2020 (1). The characteristic of this virus is that it can be transmitted through human contact. Multiple prevention strategies were instated including, social distancing, quarantine, and school closures, and as a result, people became isolated (2). Moreover, people's work and lifestyles changed from offline to online, resulting in social gatherings taking place through video chat and online education becoming a larger part of education (3). During this period, there have been steady reports of increased game use (4–7).

During COVID-19, people who become socially isolated and inactive experience high levels of stress and various mental health problems (8, 9). Additionally, previous studies found that individuals with higher levels of depression, anxiety, and stress were more likely to develop an internet game disorder (10–14). As previously existing ways to relieve stress were impeded due to COVID-19, an increasing number of individuals began to relieve stress through online games and disconnect from reality. Therefore, the phenomenon of increased gaming behavior was reasonable during this period (15).

Particularly, during COVID-19, there have been higher levels of anxiety, depression, irritability, hyperactivity, inattention, sleep disturbances, and various mental health problems in children and adolescents (16–20). A growing body of research has found that such problems may lead increase vulnerability to internet and game addictions (21).

It is known that children and adolescents become independent of their parents, gradually forming self-identity and relieving stress through relationships with peers (22). As social connection and emotional support are critical at this developmental stage (23), adolescents tried to connect with their peers online during the COVID-19 pandemic (24). Therefore, a rapid increase in the internet, social media, and game usage time of socially isolated adolescents was reported during the social distancing period of COVID-19 (15, 18, 24–26).

On the other hand, some positive aspects of games (e.g., stress relief, social connections between isolated individuals) were found in previous studies (21, 27, 28). The WHO encouraged individuals who were isolated in their homes to play games as a way to relieve stress in the early days of the pandemic (26). From this aspect, it might be a reasonable approach for people to relieve stress by playing games excessively during the pandemic. However, relieving negative emotions through this type of behavior not only strengthens the behavior but also makes it habitual (29–33). Furthermore, excessive gaming and game addiction may result in a wide variety of emotional and behavioral problems, including social isolation in the relationships of adolescents (e.g., family, friends).

Pathological game use is defined in two ways. First, the Diagnostic and Statistical Manual of Mental Disorders 5 (DSM-5) describes the following symptoms as criteria for internet gaming disorder as a condition warranting further study: preoccupation, withdrawal, tolerance, failure to reduce or stop gaming, neglect other activities, continuing gaming despite problems, deception, gaming to escape, and relationship risk due to excessive gaming (34). A diagnosis of internet gaming disorder (IGD) requires experiencing five or more of these symptoms within a year. Second, gaming disorder (GD), which is classified by the 11th edition of the International Classification of Disease (ICD-11), requires the following symptoms: impaired control, increased priority given to gaming, and continued gaming despite negative consequences. A diagnosis of GD requires three symptoms within a year (35). As such, since there is inconsistency in the diagnostic criteria and evidence of gaming disorder, there is a need to conduct more research in this area and have a comprehensive perspective on the studies that have been conducted thus far.

Additionally, as seen from the DSM-5 diagnostic criteria, IGD is used interchangeably with problematic internet usage clinically, and related research has been generally conducted together. However, problematic internet use and problematic internet gaming have different clinical characteristics (36) and physiologically exhibit different mechanisms in the brain (37). Moreover, it might be more appropriate to specifically use the term (i.e., game addiction) to conduct research instead of broadly using the term (i.e., media addiction) to better understand the mechanism of addiction (32).

In a previous study, Masaeli et al. (38) synthesized the prevalence of addiction for overall internet addiction, which increased during COVID-19 in 2021, and gave insight into the relationship between COVID-19 and game addiction. However, they focused on all age groups, and did not show the specificity of children and adolescents, and therefore the results presented in the article have limitations in showing only the prevalence of addiction in general. In addition, since the study was conducted only 8 months after the outbreak of COVID-19, it will be meaningful to collect additional studies and conduct the relevant study again.

### Research aims

During the COVID-19 period, socially isolated children and adolescents were under significant stress because they did not receive the appropriate support that they should receive through peer relationships (22). It seems that these stresses made children and adolescents vulnerable to game addiction (21). For this study, we limited the groups to children and adolescents, who are vulnerable populations, and conducted a systematic review referring to various literature on the effects of COVID-19 on digital addiction, especially game addiction. We investigated not only the prevalence rate of gaming disorder in children and adolescents but also how various factors experienced by children and adolescents affected gaming disorder during the COVID-19 period. Therefore, in this article, we tried to clarify the following questions: *Did the prevalence of game addiction among children and adolescents increase during the COVID-19 period?* (Research Question 1)

In addition, what makes children and adolescents different from adults in clinical practice is that they are not independent and their lives are influenced by the primary caregiver(s) (22). Therefore, as a second question, we wanted to know: *How did the relationship with caregivers affect the gaming disorder of children and adolescents?* (Research Question 2)

Additionally, children and adolescents experience unique stressful situations compared to those experienced by adults. Therefore, as a third question, we wanted to answer: *How did the special stress experienced by children and adolescents affect their gaming disorder?* (Research Question 3)

Finally, we *investigated game addiction during COVID-19 in children and adolescents with preexisting psychiatric diseases, who are more vulnerable*. (Research Question 4)

## Methods

Our review adhered to the steps described in the Preferred Reporting Items for Systematic reviews and Meta-Analyses (PRISMA) statement (39).

### Data sources and search strategy

We searched PubMed, Embase, psycINFO, and Cochrane on 5 May 2021 to identify relevant literature on game addiction during the COVID-19 pandemic. We used search items related to gaming disorder (game OR game addiction OR gaming OR gaming disorder OR internet gaming disorder) and COVID.

### Study inclusion and exclusion criteria

Articles were included if: (1) there was a quantitative scale to measure game addiction, (2) the research was conducted after the COVID-19 outbreak, (3) the target group of research was children or adolescents (under 19 years old), and (4) it was written in English. In addition, the criteria for exclusion in the process of selecting data were as follows: (1) the target of studies was over 18 years old, (2) studies measured only internet addiction, smartphone addiction, or screen addiction, not game addiction, (3) studies only covered the pre-COVID-19 period (we included studies comparing the periods before and after the outbreak of COVID-19, together), or (4) they were not empirical studies.

### Study selection

Articles were included in the study through the following process. First, by reading the titles and abstracts of the searched articles. Studies that fit the topic to be addressed in our research were selected by referring to the inclusion and exclusion criteria. Next, the entire texts of the selected articles were read, and those that met the inclusion criteria were selected.

### Quality assessment

The quality of the study was synthesized by two researchers independently using the Grading of Recommendations, Assessment, Development and Evaluations (GRADE) approach (40). As in the previous study that conducted a systematic review using this tool (38), we classified the research into four categories and recorded the quality of evidence for each study in Table 1 as follows: high (the authors have a lot of confidence that the true effect is similar to the estimated effect), moderate (the authors believe that the true effect is probably close to the estimated effect), low (the true effect might be markedly different from the estimated effect), and very low (the true effect is probably markedly different from the estimated effect).

**Table 1:** Description of the included studies.

**Author, Year, Country**	**Title**	**Type**	**Objecitive**	**Methodology**	**Scale**	**Poulation and age**	**Conclusion**	**Certainty in evidence**
Chen et al. (2021) China (42)	Internet-related behaviors and psychological distress among schoolchildren during the COVID-19 school hiatus	Original research	To evaluate levels of problematic gaming, problematic social media use, and problematic smartphone use; distress; and time spent on different activities; and test the mediating roles of problematic gaming, problematic social media use, and problematic smartphone use in the associations between psychological distress and screen time use	Quantitative (school-based survey)	IGDS-SF9_1)_	*N* = 2,026 Mean age = 10.71	From the aspect of practice, parents and caregivers need to monitor the use of Internet-related activities of their children while finding ways to facilitate the time spent on exercise and studying, which may contribute to better mental health among their children	High
Chen et al. (2021), China (42)	Problematic internet-related behaviors mediate the associations between levels of internet engagement and distress among schoolchildren during COVID-19 lockdown: A longitudinal structural equation modeling study	Original research	To assess changes in the level of engagement in internet-related activities before and during the COVID-19 outbreak; to investigate the differences of psychological distress before and after COVID-19 outbreak; and to investigate the mediating roles of problematic internet-related behaviors in the causal relationships of psychological distress and time spent on internet-related activities	Quantitative (school-based survey)	IGDS-SF9_1)_	*N* = 535 Mean age = 10.32	Increased problematic use of internet-related activities among schoolchildren was associated with greater psychological distress. Parents should therefore monitor internet-related activities and psychological distress of their children to support their mental health	Moderate
Cuong et al. (2021), Vietnam (43)	Associations between gaming disorder, parent-child relationship, parental supervision, and discipline styles: Findings from a school-based survey during the COVID-19 pandemic in Vietnam	Original research	To assess the prevalence of GD among Vietnamese adolescents in Hanoi, and; to assess the associations between parent-child relationship, parental discipline styles, and GD	Quantitative (school-based survey)	IGD-20_2)_	*N* = 2,084 Mean age = 14.5	“We found associations between gaming disorder and parent-child relationship, parental supervision, and parental discipline. Future interventional studies should consider assessing the effect of fostering healthy	Moderate
							parent-child relationships and appropriate discipline on the occurrence or prognosis of gaming disorders	
De Pasquale et al. (2021), Italy (44)	Online videogames use and anxiety in children during the COVID-19 pandemic	Original research	To assess the prevalence of videogames use and addiction in a sample of Italian children during the COVID-19 pandemic and their association with anxiety symptoms	Quantitative (school-based survey)	VASC_3)_	*N* = 162 Mean age = 9.4	Recently, a possible use of active video games for improving mental health and physical fitness during isolation periods was reported; however, more studies are needed to define the most adequate interventions to be activated by caregivers to prevent the negative consequences and maximize the developmentally positive effects of videogames	Moderate
Donati et al. (2021), Italy (45)	Gaming among children and adolescents during the COVID-19 lockdown: the role of parents in time spent on video games and gaming disorder symptoms	Original research	To analyze video gaming habits in children and adolescents during the lockdown, starting in March 2020 in Italy, the first European country affected by the pandemic. Specifically, we aim to understand how variables related to parents are related to their offspring's time spent on video games and GD symptoms	Quantitative (web survey)	VGS-P, VGS-A, VGS-C_4)_	Children (*N* = 206, Mean age = 8.62) Adolescents (*N* = 248, Mean age = 14.17)	Especially in this pandemic period, parents must provide alternative avenues for social interaction between adolescents in order to maintain their learning motivation and to monitor and regulate their gaming time, thus minimizing addiction risks	Moderate
Elsayed (2021), United Arab emirates (46)	Covid-19 pandemic and its impact on increasing the risks of children's addiction to electronic games from a social work perspective	Original research	To determine the impact of the Covid-19 pandemic on increasing the social, psychological, behavioral, and health risks of children's addiction to electronic games from a social work perspective	Quantitative (online survey)	Questionnaire designed by researcher on the risks of child addiction to electronic games	*N* = 289 Mean age = unknown (range = 6–17 years)	It was clearly noticed that after the Covid-19 pandemic and children staying at home for long periods of time, The rates of risks (social - psychological - health - behavioral) of children's addiction to electronic	Low
							games in its various forms have increased, especially violent games	
Fazeli et al. (2020), Iran (29)	Depression, anxiety, and stress mediate the associations between internet gaming disorder, insomnia, and quality of life during the COVID-19 outbreak	Original research	To examine the mediating role of psychological distress in the association between internet gaming disorder and two health outcomes among adolescents during this COVID-19 pandemic	Quantitative (web survey)	IGDS9-SF_1)_	*N* = 1,512 Mean age = 15.51	IGD is associated with different psychosocial outcomes comprising multiple pathways. Parents need to pay special attention to how much time and how frequently their children play videogames. Parents may need to assist their children in coping with psychological distress during the ongoing COVID-19 pandemic period	Moderate
Kim and Lee (2021), Korea (47)	Addictive internet gaming usage among Korean Adolescents before and after the outbreak of the COVID-19 pandemic: a comparison of the latent profiles in 2018 and 2020	Original research	To explore the different profiles of addictive internet gaming behavior among adolescents before and after the outbreak of the COVID-19 pandemic and examine how the pandemic influenced addictive internet gaming usage and time spent playing games on the internet	Quantitative (nationally representative survey data)	MGUS_6)_, average gameplay time	*N* = 3,040 Mean age = 13.46 in 2018 *N* = 2,906 Mean age = 13.62 in 2020	Although the results of the present study indicate that profiles with higher addictive internet gaming usage exhibit longer gameplay time, caution should be exercised when interpreting higher gameplay time as problematic. [...] Most significantly, playing games online should not be stigmatized as gaming is not pathologic and it does have positive effects. In addition, games can be utilized in educational purposes	Moderate
Kim et al. (2021), Korea (48)	Latent profile of internet and internet game usage among South Korean adolescents during the COVID-19 pandemic	Original research	To investigate the latent profiles of the Internet and Internet game usage among adolescents in South Korea	Quantitative (secondary data obtained from a national survey)	MGUS_5)_	*N* = 2,984 Mean age = 13.6	Profiles with higher game usage time scored higher in problematic game use compared to other profiles. Males were more likely to be in the profiles with high gaming time, and females were more likely to be in Internet and Smartphone	Moderate
							User profiles. The results indicate that Internet and Internet gaming usage patterns could be classified by the type of device used and the content of the Internet	
Ko and Yen (2020), Taipei (49)	Impact of COVID-19 on gaming disorder: Monitoring and prevention	Commentary	To assert that mental health professionals should be aware of how increased gaming during the pandemic may contribute to risk of gaming disorder	N/A	N/A	N/A	Parents and educators must provide alternative avenues for social interaction among adolescents in addition to maintaining their learning motivation and monitoring and regulating their gaming time, all of which could be essential to minimizing GD-related risks during this pandemic. Furthermore, mental health professionals must provide emotional support and advice on coping strategies to relieve pandemic-related stress in individuals	–
Li et al. (2021), Canada (3)	Screen use and mental health symptoms in Canadian children and youth during the COViD-19 pandemic	Original research	To determine whether specific forms of screen use were associated with symptoms of depression, anxiety, conduct problems, irritability, hyperactivity, and inattention in children and youth during COVID-19	Quantitative (longitudinal cohort study)	Video game time	Group 1 *N* = 532 Mean age = 5.9 Group 2 *N* = 1,494 Mean age = 11.3	In this cohort study, higher levels of screen use were associated poor mental health of children and youth during the COVID-19 pandemic. These findings suggest that policy intervention as well as evidence-informed social supports are needed to promote healthful screen use and mental health in children and youth during the pandemic and beyond	Moderate
Oliveira et al. (2021), Brazil (50)	Children's behavioral problems, screen time, and sleep problems' association with negative and positive parenting strategies during the COVID-19 outbreak in Brazil	Original research	To investigate the group differences among children raised by negative and positive parenting families during COVID-19 pandemic.	Quantitative (online survey)	GAS_6)_	*N* = 329 Mean age = 10.24	Children and adolescents might have an amplified impact during pandemic depending on the parenting strategies mostly used. Considering parental management training is an effective strategy to improve parenting strategies and it is available even online, it might consist of ground to have a potential improvement in developmental competencies and in children and adolescent's mental health even during pandemic times	Moderate
She et al. (2021), China (51)	How COVID-19 stress related to schooling and online learning affects adolescent depression and Internet gaming disorder: Testing Conservation of Resources theory with sex difference	Original research	To test the roles of stress related to schooling and online learning during COVID-19 in depression and IGD among adolescents and the potential mediators of social support, academic stress, and maladaptive emotion regulation based on the framework of Conservation of Resources theory	Quantitative (school-based survey )	DSM-5 IGD Symptoms checklist	*N* = 3,136 Mean age = 13.6	Although stress and disruptions to daily life are inevitable during the pandemic, psychosocial interventions and preventive measures targeting these modifiable mediators have the potential to help reduce the risk of depression and IGD and facilitate students to adapt to the COVID-19 era	Moderate
Shuai et al. (2021), China (52)	Influences of digital media use on children and adolescents with ADHD during COVID-19 pandemic	Original research	To explore the influences of digital media use on the core symptoms, emotional state, life events, learning motivation, executive function and family environment of children and adolescents diagnosed with ADHD during the (COVID-19) pandemic	Quantitative	Average hours on games	ADHD patient *N* = 192, Mean age = 11.02	The ADHD children with problematic mobile phone use (PDMU) suffered from more severe core symptoms, negative emotions, executive function (EF) deficits, damage on family environment, pressure from life events, and a lower motivation to learn.	Low
							Supervision of digital media usage, especially video game and social media, along with increased physical exercise, is essential to the management of core symptoms and associated problems encountered with ADHD	
Teng et al. (2021), China (53)	Depression and anxiety symptoms associated with internet gaming disorder before and during the COVID-19 pandemic: A longitudinal study	Original research	To examine gaming in the context of the pandemic and its association with depressive and anxiety symptoms	Quantitative (longitudinal study)	IGDS9-SF_1)_	*N* = 1,778 Mean age = unknown(children and adolescents)	Children and adolescents both increased videogame use during the COVID-19 pandemic, but only adolescents significantly increased IGD severity during the COVID-19 pandemic. The findings supported the compensatory hypothesis, and are consistent with the Interaction of Person-Affect-Cognition-Execution model as individual responses to COVID-19 may function as a mediator between personal predisposing variables and IGD	Moderate
Wang et al. (2022), China (54)	Anxiety, depression and stress are associated with internet gaming disorder during COVID-19: fear of missing out as a mediator	Original research	To explore whether the difference exists in the relationship between depression, anxiety, or stress and Internet gaming disorder, and to explore how fear of missing out influences depression, anxiety, or stress	Quantitative (school-based survey)	IGDS_7)_	*N* = 324 Mean age = 13.07	The results indicated that fear of missing out as a mediator regulates the relationship among depression, anxiety, and stress and Internet game disorder.	Moderate
							Specifically, under the mediation of fear of missing out, teenagers with anxiety are more likely to develop Internet gaming disorder, while teenagers with depression or stress might be prone to other types of Internet use disorders	
Wu et al. (2022), China (55)	Changes of internet behavior of adolescents across the period of COVID-19 pandemic in China	Original research	To describe the internet behavior changes of adolescents and to understand the impact of clinical features on internet addiction after the adolescents back to school in COVID-19 period	Quantitative (cross-sectional cohort study through online survey)	Internet gaming behaviors	*N* = 625 Mean age = 14.90	There are differences in the clinical characteristics between the adolescents with and without Internet addiction. When intervening in adolescents' problematic Internet behavior during the COVID-19 pandemic, the heterogeneity in characteristics between subgroups should be considered	Low
Zhu et al. (2021), China (28)	Leisure and problem gaming behaviors among children and adolescents during school closures caused by COVID-19 in Hong Kong: quantitative cross-sectional survey study	Original research	To examine the associations between loneliness and gaming addiction behaviors among young people in Hong Kong and to investigate how familial factors, psychological distress, and gender differences moderate these relationships	Quantitative (cross-sectional study)	GAS_6)_, Gaming Time	*N* = 2,863 Mean age = 12.6	Loneliness was associated with gaming addiction behaviors; the findings from this study suggested that this association was similar across gender and age groups among young people. Familial support and supervision during school closures can protect young people from developing problematic gaming behaviors. Results of this study have implications for prevention and early intervention on behalf of policy makers and game developers	Moderate

*1) Internet Gaming Disorder Scale-Short Form (IGDS-SF9), 2) Internet gaming disorder Test (IGD-20), 3) Videogame Addiction Scale for Children (VASC), 4) Video Gaming Scale for Parents, Adolescents and Children (VGS-P, VGS-A, VGS-C) 5) Maladaptive Game Use Scale (MGUS), 6) Game Addiction Scale (GAS) 7) Internet Gaming Disorder Scale (IGDS)*.

### Data extraction and synthesis

Data extraction included the following: year, country, mean age, title of article, research type, objective and results of studies, scale to define game addiction, and gaming hours. As the first question of our study was to measure the global prevalence of game addiction during the pandemic, we extracted prevalence estimates of game addiction from the studies. For studies that did not have prevalence estimates but presented gaming hours, we considered gaming more than 5 h as game addiction (41). Then, we read the articles included in the study several times, found the answers to the questions raised, and synthesized them into several themes. Afterward, we reviewed the synthesized themes to derive the final result.

## Results

### Study characteristics

**Figure 1:**
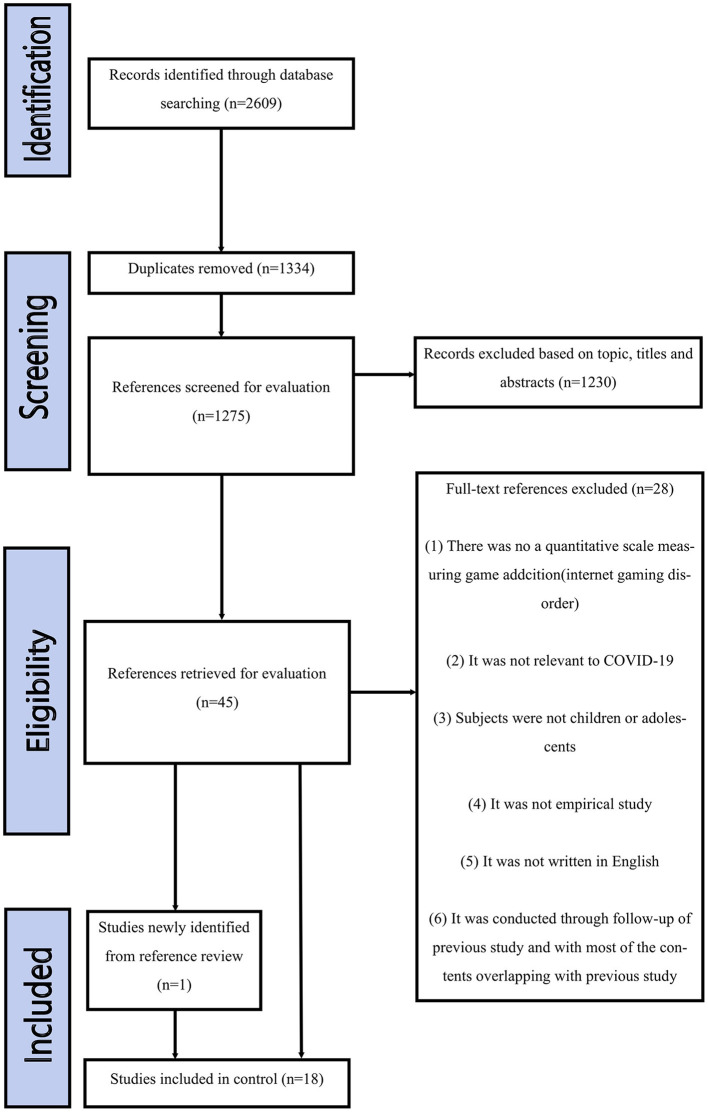
Flow chart of the search strategy and study selection.

We collected studies that were published between June 2020 and February 2022, which was during the pandemic. We identified 2,609 articles. Among them, studies that were not related to the topic, duplicated, conducted through follow-up of previous studies and with most of the contents overlapping with previous studies, and that did not meet the selection criteria were excluded, and 18 studies were selected (Figure 1). Among the 18 studies, there were two studies in 2020, 14 studies in 2021, and two studies in 2022. The majority of studies were original articles. However, one study conducted by Ko et al. (49), which was a commentary, was included as an exception because they played a key role in the overview and review of this study. Although we aimed to include studies that had valid scales, there are a wide variety of valid scales used for GD, which are the internet Gaming Disorder Scale-Short Form (IGDS-SF9), internet Gaming Disorder Test (IGD-20), Videogame Addiction Scale for Children (VASC), Video Gaming Scale for Parents (VGS-P), Video Gaming Scale for Adolescents (VGS-A), Video Gaming Scale for Children (VGS-C), Maladaptive Game Use Scale (MGUS), Game Addiction Scale (GAS), internet Addiction Test (IAT), and DSM-5 IGD Symptoms checklist (Table 1).

As described above, the scales used for game use varied greatly depending on the study, and the definition of pathological game use was also different, so the main results of the studies were heterogeneous. The age range of the subjects also differed from study to study. Therefore, we did not perform statistical meta-analysis due to concerns that differences in study characteristics would materially affect the results. Instead, we descriptively present the prevalence rates suggested by the studies to determine the difference between the prevalence before and after COVID-19.

### Quality assessment

Of 18 studies, 14 studies were evaluated as moderate, three studies were evaluated as low, and one study was evaluated as high. Most of the studies were large-scale studies, presenting statistically significant results. We concluded that these studies were evaluated as moderate or low due to their study design (i.e., observational studies).

We reviewed the studies and summarized the conclusions of our previous questions into four themes, which are as follows. (1) Changes in the prevalence of game addiction among children and adolescents during COVID-19. (2) Impact of the relationship with parents on gaming disorder in children and adolescents. (3) Effects of specific stress experienced by children and adolescents on gaming disorder during COVID-19. (4) Effect of the Vulnerable population and COVID-19 on gaming disorder. In addition, reviewing the studies, we found that the prevalence of game addiction and factors influencing it were different according to age and gender, so we added, (5) Gaming disorder according to demographic characteristics.

### Changes in the prevalence of game addiction among children and adolescents during COVID-19

The impact of COVID-19 on gaming disorder was examined in two aspects: whether game usage time increased and whether game addiction (e.g., IGD) scores measured on a game addiction scale increased during COVID-19. Several studies found increased game usage time and increased levels of game addiction (42, 47, 48, 56). Among 18 articles, seven presented prevalence estimates of game addiction using scales, and three additional articles presented specific gaming hours. In a total of 10 articles, the prevalence estimates of game addiction that were measured by each study are shown in Table 2. Many studies found that the prevalence of gaming disorder increased, which was estimated to be between 2.3 and 29.4%, and the global prevalence of IGD was 1.96% before the pandemic (43, 45–48, 51, 56). However, there was difficulty demonstrating comprehensive results, as the studies used inconsistent scales, defined addiction differently, and had differences in the prevalence estimates of addiction. Therefore, an agreement on the scale of game addiction should be made through continuous research in this area.

**Table 2:** Prevalence of game addiction according to studies.

**Studies**	**Scale**	**Game addiction (%)**
Kim D. J. et al.	MGUS	2.3
Teng et al.	IGDS-SF9	5
Cuong et al.	IGD-20	11.6
Zhu et al.	N/A^*^	13.6
She et al.	IGDS-SF9	14.8
Kim et al.	MGUS	16.1
Li et al. adolescent group	N/A^*^	17.8
Donati et al. child group	VGS	19
Donati et al. adolescent group	VGS	22
Chen, C.Y. et al.	IGDS-SF9	23.7
Elsayed et al.	N/A^*^	29.4

*MGUS, Maladaptive Game Use Scale; IGDS-SF9, Internet Gaming Disorder Scale-Short Form; IGD-20, Internet gaming disorder Test; VGS, Video Gaming Scale; N/A, not available*.

* *More than five gaming hours were considered as Game addiction in the present study*.

Many of these studies had a limited study design (i.e., cross-sectional), as they investigated the impact over the limited period of COVID-19. To better investigate its causality, several studies attempted to obtain and examine temporality. Kim and Lee demonstrated that there was a significant increase in game usage time and game addiction during the COVID-19 pandemic by comparing a group that was recruited before COVID-19 with a group that was recruited during COVID-19 (47). In a study conducted in China, students who returned to school after the pandemic were asked to retrospectively report their game usage time and measured their game addiction scores over three different periods (i.e., before, during, and after COVID-19). After their scores were compared, the study found an increase in game usage time and game addiction score that were measured during the pandemic. Moreover, there was a cohort study that demonstrated an association between game time and game addiction score in its cohort group that was compared before and after COVID-19 (3, 53). However, further investigation with different types of groups should be continued, as there are small sample size studies that did not find an increase in game addiction (44, 57).

There have been inconsistent findings on the impact of games on mental health since before COVID-19 (58–66). Many studies have investigated the increased use of games and their effects on mental health during the pandemic. A majority of the studies found a positive correlation between game usage time and game addiction score (45–48). Li et al. (3) demonstrated temporal causality between game usage time and game addiction score by measuring game usage time and game addiction scores of a cohort group of 2,026 participants several times. Moreover, such a correlation increased levels of depression, anxiety, stress, fear of missing out, irritability, inattention, hyperactivity, and hyperactivity/impulsivity, which exhibited the effects of game addiction on mental health (3, 46, 48, 50, 54, 55, 57).

### The impact of relationship with parents on gaming disorder in children and adolescents

During the COVID-19 pandemic, there has been a large body of research regarding the impact of parenting on gaming disorder (43, 45, 50, 56). Zhu et al. (56) found that perceived parental supervision played a role in reducing pathological gaming behavior. However, parental support played a role as a protective factor against an increase in game usage time only in primary school students but played a role as a risk factor in secondary school students. Another study in Italy (45) also demonstrated that children's GD symptoms were negatively related to parental video gaming monitoring and parental knowledge of them. These studies demonstrated a perspective that supports parental care and intervention for children.

Some studies more specifically examined parenting behavior (43). For instance, interestingly, the lowest prevalence of GD was shown in participants who were under parental supervision, whereas a high prevalence of GD was shown in those under supervision with severe physical punishment, those under supervision without discipline, and those with no parental supervision. That is, violent parenting increased levels of game addiction in children, but the absence of parental supervision also increased them. However, as the authors revealed, this should be taken into account cautiously to reveal causal inference, considering the possibility that harsh parenting might have occurred due to game addiction of their offspring. From a different perspective, a study focused on parenting style was conducted. Oliveira et al. (50) investigated and measured authoritarian and authoritative parenting styles and their relationship with children's mental health behavior, including gaming behavior. As a result, the children of authoritarian parents reported significantly higher internalizing and externalizing symptoms, excessive game usage time, and game addiction scores. In particular, regulation and punishment were significantly associated with game addiction, and all behavioral problems were associated with less autonomy, physical coercion, and verbal hostility. As the authors highlighted, the regulation used to control behavioral problems fostered the problems

### Effects of specific stress experienced by children and adolescents on gaming disorder during COVID-19

Some studies found that game usage time was increased by the social needs of isolated children and adolescents during the COVID-19 pandemic. Among them, Wang et al. focused on the fear of missing out (54). Fear of missing out is a pervasive apprehension that others might be having rewarding experiences from which one is absent, characterized by the desire to stay continually connected with what others are doing. They demonstrated that depression, anxiety, and stress in children and adolescents were linked to internet gaming disorder through the mediating effect of “fear of missing out.” This relationship was shown more significantly in anxiety. That is, children and adolescents in emotional pain, play games to communicate with their peers to not be separated from them. In another study (56), loneliness was quantitatively associated with excessive and pathological gaming behavior. Even after other factors were adjusted, the odds ratio remained increased. The authors of the study indicated that children and adolescents play games to feel less lonely and connect with peers, as there were fewer opportunities to meet friends, which also aligns with some findings that loneliness is associated with psychiatric symptoms during COVID-19 among children and adolescents (20).

Additionally, one study mainly focused on psychosocial factors of adolescents that were associated with COVID-19 (51). The authors exhibited a correlation between COVID-19 stress related to schooling and online learning and IGD in adolescents with an average age of 13.6. They also found mediating effects of social support, academic stress, and emotional regulation on the relationship between COVID-19 and IGD. That is, adolescents faced problems with social support, academic stress, and emotional regulation through online learning, which led to the emergence of IGD problems. In particular, emotional regulation showed the highest correlation, which aligns with some previous findings that displayed the effects of loneliness and depression on problematic internet use in a group of deficient self-regulation (31).

### Effect of the vulnerable population and COVID-19 on gaming disorder

During the COVID-19 period, there have few studies regarding game addiction in high-risk groups. Shuai et al. (52) demonstrated increased video game time in patients who were previously diagnosed with attention-deficit/hyperactivity disorder (ADHD) during the COVID-19 pandemic, while children with game and digital use problems showed worsened existing ADHD core symptoms, executive functions, and oppositional systems, as well as worsened emotional problems in their familial relationships. Meanwhile, a longitudinal study with 1,778 subjects found that levels of depression and anxiety in the pre-COVID-19 period increased the prevalence of internet gaming disorder during the COVID-19 period (53). This result remained significant even when the impact due to COVID-19 was set as a mediator. With regard to the result, it might be possible that behavioral addiction, such as IGD, was caused by the impact of COVID-19 in the vulnerable group with predisposing factors. On the other hand, according to one study with a sample of 162 children, the current anxiety state was associated with games but not with trait anxiety. Although this did not coincide with previous findings, it is important to note that it was cross-sectional and limited to a small sample in one area, resulting in some limitations to its interpretation (44).

### Gaming disorder according to demographic characteristics

The demographic composition that could affect the association between COVID-19 and gaming disorder was split into age and gender in the present study. Regarding age, both children and adolescents reported increased game usage time (45, 53), but adolescents scored higher on a game addiction scale (45, 53, 56). From the aspect of parenting, children were more likely to be cared for by their parents, have higher monitoring scores, and control their game usage time better (45, 56). As independence is an important issue for adolescents (67), this could be seen as an advantage that younger children who are less independent cooperate well with their parents (53). Additionally, unlike children, loneliness was associated with pathological gaming in adolescents, which indicates that the need to use games might differ based upon age (56). The difference in results according to the age of a target group in each study was not quantitatively corroborated, as various confounding factors could influence the results and their gaming usage scales were heterogeneous. Therefore, it may be necessary for future studies to unify gaming scales and conduct a systematic review including statistical analysis such as meta-regression based on the influence of age, controlling for all possible confounding factors.

Regarding gender, males reported longer game usage time (44, 45, 47, 48, 51, 56) and higher scores of game addiction than females (45, 47, 48, 51, 56). As loneliness played a significant role as a mediator (56) in females with higher scores of game addiction and increased scores of game addiction were reported in those who excessively used smartphones, the mechanism of using games might be different based on gender. Moreover, with regard to parenting, females had a higher degree of parental supervision than males, which might have served as a protective factor (45).

## Discussion

This review aimed to determine the effects of COVID-19 on gaming disorder among children and adolescents. Although similar prevalence studies have been conducted (38), we limited the group to children and adolescents and wanted to take a deeper look into how their actual experiences during COVID-19 were related to gaming disorder. We reviewed 18 studies and synthesized five themes: (1) Changes in the prevalence of game addiction among children and adolescents during COVID-19, (2) The impact of the relationship with parents on gaming disorder in children and adolescents, (3) Effects of specific stress experienced by children and adolescents on gaming disorder during COVID-19, (4) Effects of Vulnerable population and COVID-19 on gaming disorder, and (5) Gaming disorder according to demographic characteristics.

Peer relationships and relationships with parents both play an important role in the formation of self-identity and socially healthy development of children and adolescents (22). During the COVID-19 period, isolation from social relationships such as school and frequent conflicts with parents affected the use of games, which varied according to gender, age, and preexisting vulnerabilities.

Regarding the change in prevalence of gaming disorder during COVID-19, consistent with conclusions from previous studies on the general population (38), the proportion of groups included in gaming disorder among children and adolescents increased in most studies. Children and adolescents showed a decrease in gaming disorder with appropriate supervision from parents, but the lack of supervision or harsh discipline increased gaming disorder in children and adolescents, contrary to the parents' intention. During COVID-19, children and adolescents experienced increased stress in peer relationships, which manifested as a fear of missing out, a lack of social support due to online learning, and a feeling of loneliness, which affected gaming disorder. These effects were more severe in the group with ADHD or existing psychiatric symptoms, and gaming disorder inversely worsened the core symptoms of ADHD. Adolescents, in which peer relationships are more important than children, showed a higher game addiction scale due to emotional problems experienced during COVID-19, but they had difficulty receiving supervision by their parents due to their unique preference for independence. In terms of gender, although males overall scored higher in game addiction, female loneliness acted as a more important mediator for game use. Therefore, it was judged that there was a difference in game use according to gender.

### Overcoming gaming disorder during the COVID-19 pandemic in children and adolescents

The COVID-19 pandemic continues today, children and adolescents are constantly exposed to stress, and the resulting gaming use disorder continues. Therefore, based on the results found in this study, we intend to examine and find ways to overcome the current situation.

The most important aspect to pay attention to is parenting. Due to social quarantine since the COVID-19 pandemic occurred, social institutions, including schools, were locked down. As a result, parents took on a large part of the supervision that schools would have taken charge of. Since parents spend more time with their children at home as they work from home, the role of parents has become more important. This might explain why many studies examined the role of parents since the pandemic occurred (45). However, during COVID-19, parents have not been well aware of the excessive gaming of their children and have provided supervision well due to their attention to the prevention of COVID-19 and the economic and social effects caused by the pandemic (49).

As seen in the studies, parents are to appropriately guide their children by recognizing their game patterns, helping them control their game usage time, and monitoring their game usage time, since their knowledge of their children serves as a protective factor to develop socially and psychologically (46).

Kiraly et al. (33) published consensus guidance on internet use disorder during COVID-19 in a group that included psychologists and psychiatrists from the United States and several European countries. They recommend monitoring screen time within the guidance and, in particular, regulating children's behavior for rule-making. As in the study conducted by Oliveira et al. (50), authoritative parenting styles, rather than coercive parenting, acts as a protective factor for gaming disorder. Therefore, it seems necessary for parents to maintain their authority but to reach an agreement on game use through rational dialog with their children. As a way to help with this, apps that give feedback on game time seem to be helpful (33).

Király et al. also advised in the consensus guideline mentioned above that by participating in the games that their children play together, parents can learn more about the games their children are playing and help control their children's games (33). Play itself is not harmful but rather helpful for their overall development. Moreover, since play is part of their lives, they accomplish developmental tasks, grow mentally, and relieve mental stress (22). As parents become familiar with their children's games, they will be able to assist their children in avoiding killing or assaulting games, but rather playing age-appropriate games (50, 68). Additionally, parents should choose good games such as educational electronic games or exergame or different activities such as table games, home exercise, and reading (46).

In a study conducted in a region of China during COVID-19, a group with increased reading, studying, or exercising time reported decreased internet-related behavioral problems such as games (42). Accordingly, fostering and encouraging other activities may be one of the strategies to reduce game addiction (45). Furthermore, considering the finding that the game usage time of parents increased the game usage time of their children, we suggest that parents decrease their game usage time and try not to play games in front of them as much as possible (56).

Additionally, there is an association between hostile and less supportive parenting and the stress levels of parents (69, 70). Negative parenting, such as less autonomy, verbal hostility, physical coercion, and punishment, affects different externalizing symptoms, including game addiction in children (43, 50). Taken together, mental health providers should provide parents with emotional support to prevent them from burning out and teach them supportive parenting techniques.

Intervention in paradoxical situations experienced by adolescents is also important. Although adolescents are generally vulnerable to game addiction, unfortunately, such aforementioned strategies were less effective or had adverse effects on this population in several studies regarding parenting (53). As mentioned above, as independence is critical in adolescence, the supervision of parents seems to be less effective (45), and they tend to be less aware of their offspring. Additionally, emotional regulation, which plays a critical role in game addiction, is relatively less developed than other cognitive functions in adolescence (51). As we have seen, lack of social support and feelings of loneliness among adolescents influenced their gaming disorder (20, 51, 54, 56). Kiraly et al. (33) reported that keeping in touch with friends, relatives, and acquaintances in consensus guidelines would help overcome these emotional difficulties. Instead of their direct interventions, parents or educators should use group calls, social media groups, or remote conferencing services to boost social support or help children reduce their academic stress so that such services could become avenues for social interaction among adolescents (33, 46).

Finally, mental health professionals should offer stress-reduction techniques (e.g., meditation, autigenic training, mindfulness exercises) that are better than using games, helping offspring and their parents stay emotionally healthy. It is also important to create a direct route to help if they encounter any issues related to using the game (33). In particular, mental health providers should provide children and adolescents who have mental health problems such as ADHD, depression, and anxiety with more intensive observations.

### Limitations

Our study has several limitations. First, our study included only studies written in English. Therefore, there is a possibility that data from various countries in the environment of writing articles in languages other than English could not be reflected. Second, we did not provide statistically specific figures. Although this was because the results of the studies included in this review were heterogeneous, we did not achieve the purpose of synthesizing the results of our study on gaming disorder in children and adolescents. Third, we tried to examine the solution to the current situation through the results of this review, but it was limited and insufficient. Further studies related to the intervention of gaming disorder are needed. Fourth, most of the included studies were cross-sectional studies, so the quality of the articles was limited. In our evaluation, most of the articles were moderate, but there were also a few articles of low quality. This is thought to be due to the nature of the studies conducted for a special limited period of time called COVID-19, but as the quality of the systemic review is determined by the included articles, it seems necessary to conduct research by collecting more high-quality studies in the future.

### Future directions

As mentioned earlier, a limitation of studies related to COVID-19 is that most of them are cross-sectional. Therefore, various high-quality studies, such as cohort studies that track changes over time or randomized controlled studies, are needed in the future. Moreover, recently, as there are articles on long COVID syndrome, the problems caused by COVID-19 are expected to continue, so it seems that research on it should be included (71).

In this review, it was confirmed that demographic characteristics affect gaming disorder. Although male and female differed in the prevalence of gaming disorders, there also appear to be differences in the purpose of gaming (56). Therefore, if studies focusing on gender differences are conducted in the future, effective interventions are likely to be made. We found that differences in developmental stages according to the age of children and adolescents affect gaming disorder. Therefore, we tried to investigate the change in prevalence according to age, but there was no correlation with an exploratory graph. This was presumed to be due to the different scales for each study and the influence of several compounding factors. Therefore, it may be necessary for future studies to unify gaming scales and then conduct a systematic review including statistical analysis such as meta-regression based on the influence of age, controlling for all possible confounding factors.

Although we suggested methods to overcome the current situation, the studies based on the proposed intervention methods were actually close to the suggestions of experts (33, 46, 50, 56, 68). Therefore, systematic intervention studies for various intervention methods should be conducted to establish the level of evidence. Additionally, in relation to COVID-19 and gaming disorder among children and adolescents, there have been few studies on psychiatric risk groups (52, 53). As we saw in our review, the psychiatric risk group was more vulnerable to gaming disorder, and the results were worsening, so it seems that research on the risk group is needed. In particular, mental health providers should provide children and adolescents who have mental health problems such as ADHD, depression, and anxiety with more intensive interventions. Therefore, it seems that various screening methods and studies to verify them will be needed in the future.

## Conclusion

The COVID-19 pandemic has had a social impact and isolated all individuals through social distancing. Isolated children and adolescents increased their gaming hours to cope with psychological pain, such as loneliness and anxiety, and avoid social isolation. As a result, internet gaming disorder has become more prevalent. Their parents who should have properly supervised them also failed to provide appropriate support due to the stress caused by the pandemic, which worsened the problem. Considering this, mental health providers should take this into account, educate children, adolescents, and their guardians on how to relieve their stress alternatively and help parents effectively control their children's usage of games. More intensive interventions should be designed for emotionally vulnerable children and adolescents.

## Author contributions

Conceptualization, project administration, resource, and supervision: M-HP. Data curation and methodology: TH, HC, and M-HP. Formal analysis, investigation, and visualization: TH and HC. Validation and writing—review and editing: TH, DS, and M-HP. Writing—original draft: TH. All authors contributed to the article and approved the submitted version.

## Conflict of interest

The authors declare that the research was conducted in the absence of any commercial or financial relationships that could be construed as a potential conflict of interest.

## Publisher's note

All claims expressed in this article are solely those of the authors and do not necessarily represent those of their affiliated organizations, or those of the publisher, the editors and the reviewers. Any product that may be evaluated in this article, or claim that may be made by its manufacturer, is not guaranteed or endorsed by the publisher.
